# Effects of high impact exercise on systemic cytokines in women with mild knee osteoarthritis: A 12-month RCT

**DOI:** 10.1016/j.ocarto.2025.100609

**Published:** 2025-04-11

**Authors:** Ville-Markus Konola, Juhani Multanen, Johanna K. Ihalainen, Jukka E. Hintikka, Timo Jämsä, Hannu Kautiainen, Miika T. Nieminen, Satu Pekkala, Maarit Valtonen, Ari Heinonen

**Affiliations:** a Faculty of Sport and Health Sciences, University of Jyväskylä, P.O. Box 35, Jyväskylä, FI, 40014, Finland; b South-Eastern Finland University of Applied Sciences, Savonlinna, Finland; c Research Unit of Health Sciences and Technology, University of Oulu, Oulu, Finland; d Medical Research Center Oulu, Oulu University Hospital and University of Oulu, Oulu, Finland; e Primary Health Care Unit, Kuopio University Hospital, Kuopio, Finland; f Folkhälsan Research Center, Helsinki, Finland; g Department of Diagnostics, Oulu University Hospital, Oulu, Finland; h Finnish Institute of High Performance Sport KIHU, Jyväskylä, Finland

**Keywords:** Osteoarthritis, MRI T2, Cytokines, Cartilage, High-impact exercise

## Abstract

**Objective:**

This study investigated the effects of a high-impact exercise regimen compared with a reference group on systemic cytokine levels in patients with mild knee osteoarthritis (OA). Furthermore, associations between cytokines and magnetic resonance imaging (MRI) transverse relaxation time (T2) mapping and metabolic equivalent task hours (MET-hours) during leisure-time physical activity (LTPA) were assessed.

**Method:**

In this secondary analysis, 73 postmenopausal women aged 50–65 years with mild knee OA were randomized to a 12-month high-impact aerobic/step aerobics training group (n ​= ​35) or a non-training reference group (n ​= ​38). The serum cytokine levels, including interleukin-1 alpha (IL-1α), IL-2, IL-4, IL-5, IL-6, IL-10, IL-13, IL-17, interferon-gamma (IFN-γ), and tumor necrosis factor alpha (TNF-α), were determined via multiplex cytokine assays. The cartilage structure of the medial tibial condyle was assessed by MRI T2 mapping. The primary outcome was between-group differences in cytokine level changes.

**Results:**

After a 12-month follow-up, no significant differences in cytokine level changes were found between the groups. In the intervention group, 12-month changes in TNF-α levels were associated with changes in medial tibial condyle T2. In the reference group, 12-month changes in IL-10 levels were associated with changes in medial tibial condyle T2 and the number of weekly LTPA MET-hours.

**Conclusion:**

A progressive high-impact exercise regimen did not affect systemic cytokine levels compared to the reference group and could therefore offer a possible mode of exercise for postmenopausal women with mild knee OA.

**Trial registration number:**

ISRCTN58314639.

## 1 Introduction

Osteoarthritis (OA) is a common cause of joint pain and functional loss, as well as a source of societal costs, in older adults [1,2]. It is a disease of unknown etiology associated with degeneration of the articular cartilage, inflammation of the synovial membrane, and changes in the subchondral bone [3,4]. In addition, local synovial inflammation is common in both the early and late stages of OA [5] and systemic low-grade inflammation has been found to be involved in the pathogenesis of OA [6,7].

Currently, there are no disease-modifying treatments available for OA; instead, treatments focus on symptom management. The core non-pharmacological interventions for OA include exercise, weight loss if appropriate, and education [8]. The first line pharmacological treatment for OA are non-steroidal anti-inflammatory drugs [8]. Furthermore, in end-stage OA with persistent pain and functional impairment, total knee replacement may be a beneficial treatment option [2]. Exercise has demonstrated positive effects on pain, physical function, and quality of life, yet no single type of exercise regimen has proven to be the most effective [[9], [10], [11]]. However, recently, the effects of therapeutic exercise on pain and physical function have been found to be small and of questionable clinical importance, especially in the medium and long term [12].

Exercise interventions do not appear to be detrimental to articular cartilage morphometry, morphology, or composition in people with or at risk of knee OA [13,14]. As OA and osteoporosis tend to affect the same population, postmenopausal women [15,16], the question has arisen as to whether favorable bone exercise, such as high-impact exercise [17,18], is safe for women diagnosed with mild OA. In our previous study, which used the same database, the findings suggested that a 12-month high-impact exercise regimen improved femoral neck bone mineral content and bone strength without altering the biochemical composition of tibiofemoral joint cartilage (transverse relaxation time (T2) and delayed gadolinium-enhanced magnetic resonance imaging of cartilage) [19,20]. In addition, the patella's cartilage quality improved [21]. Additionally, Hartley et al. [22] found that high-impact exercise regimen did not alter the biochemical composition of tibiofemoral articular cartilage between exercising and control legs, but did improve femoral neck bone mineral mass and strength in postmenopausal women. Although high-impact exercise does not appear to be detrimental to tibiofemoral cartilage in postmenopausal women with mild knee OA, it is also important to understand the effects of high-impact exercise on cytokines.

According to published reports, the levels of pro-inflammatory cytokines such as interleukin-1beta (IL-1β), IL-6, IL-15, IL-17, IL-18, and tumor necrosis factor-alpha (TNF-α), are elevated in the serum of patients with OA and act as mediators of cartilage degradation and inflammation [[23], [24], [25], [26]]. Conversely, anti-inflammatory cytokines, such as IL-4, IL-10, and IL-13, have antagonistic effects [27]. Previous studies have generally found that aerobic and strength exercise interventions do not affect long-term circulating cytokine levels in individuals with OA [28,29]. However, cytokines can also have a combination of anti-inflammatory and pro-inflammatory effects, as in the case of IL-6 [30].

However, due to the lack of data on the effects of an intensive exercise regimen on inflammatory factors in participants with mild knee OA, the primary aim of this study was to investigate the effects of a 12-month high-impact exercise regimen compared with a reference group on systemic cytokine levels in postmenopausal women with mild knee OA. In addition, secondary outcomes were assessed including the relationships between cytokine levels, knee cartilage quality using MRI T2 mapping, and weekly leisure-time physical activity (LTPA).

## 2 Materials and methods

### 2.1 Study design

The present study was a secondary analysis based on a parallel two-group randomized controlled trial, “Effects of exercise on knee cartilage and bone” (ISRCTN58314639), consisting of a 12-month impact-loading exercise intervention [[19], [20], [21]]. Study participant recruitment and data collection took place between March 2008 and April 2009. The study protocol and pre- and post-intervention results for cartilage and bone outcomes, as well as OA-related symptoms, have been reported in detail elsewhere [[19], [20], [21]]. The current study reports pre- and post-intervention results for cytokines. This study had two experimental arms: (1) a high-impact aerobic/step aerobics training group and (2) a non-training reference group. All measurements were performed at baseline before the intervention and at the end of the 12-month intervention. All outcome assessors were blinded to the treatment group allocation. All study assessments and training sessions were conducted in facilities at the Faculty of Sport and Health Sciences, University of Jyväskylä, except for the MRI imaging, which was conducted at the Central Finland Hospital Nova.

The participants were postmenopausal women aged 50–65 years with mild knee OA in the tibiofemoral joint, according to the Kellgren–Lawrence (KL) classification [31]. Specifically, they exhibited radiographic changes classified as KL I (possible osteophytes) or KL II (definite osteophytes, possible joint space narrowing) and experienced knee pain on most days. The participants were randomly assigned to groups by a statistician, who was blinded to the study participants, using a computer-generated, blocked randomization list. A block size of 10 was used and stratified according to KL grades 1 and 2. The sample size was determined by power calculations for the primary outcome of the research project [19].

The study design and reporting followed the CONSORT recommendations for conducting and reporting of randomized controlled trials [32]. The study protocol (Dnro1E/2008) was approved by the Ethics Committee of the Central Finland Health Care District. Prior to enrollment, all participants provided written informed consent in accordance with the Helsinki Declaration.

### 2.2 Participant recruitment

**Fig. 1:**
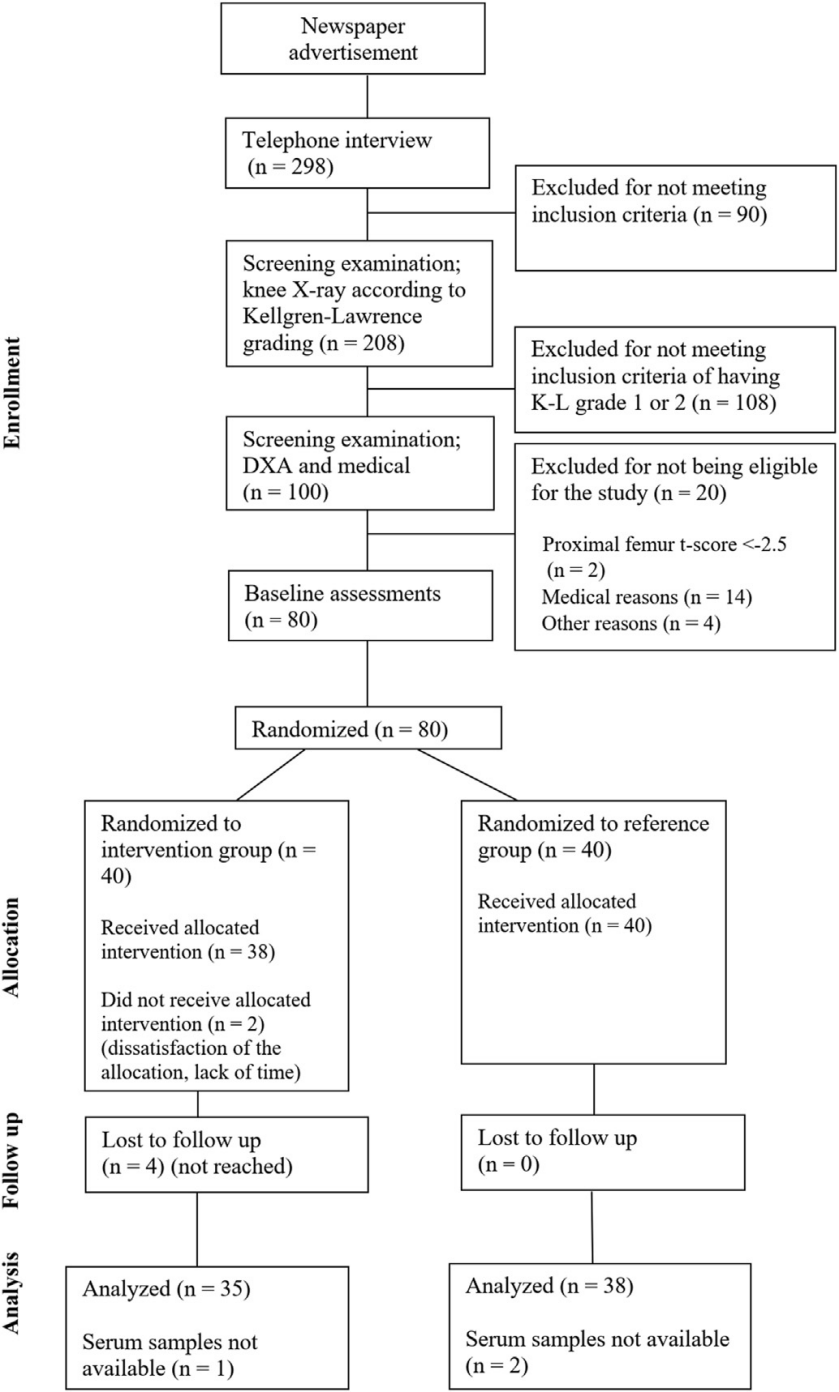
Trial profile.

Participants were recruited from the county of Central Finland using newspaper advertisements and telephone recruitment methods. A total of 298 postmenopausal women indicated interest in participation, 80 of whom met the inclusion criteria. The criteria were assessed by telephone interview, a clinical screening examination, radiographs of the tibiofemoral joints and lumbar spine, and femoral neck dual-energy X-ray absorptiometry scanning. The inclusion criteria were postmenopausal woman aged 50–65 years, who experienced knee pain almost daily, KL grades I or II, no more than two sessions of intensive exercise weekly, and no medical reason preventing full participation in intensive exercise. The exclusion criteria were a body mass index above 35 ​kg/m^2^, knee surgery or instability, osteoporosis, inflammatory joint disease, recent intra-articular steroid injections in the knee, and contraindications to MRI or contrast agents. The trial profile is presented in Fig. 1.

### 2.3 Blood sampling procedures and analyses

All measurements were performed following an overnight fast (12 ​h) with the subjects instructed to consume 0.25 ​L of water in the morning prior to visiting the laboratory. The subjects were instructed to refrain from intensive exercise for at least 48 ​h prior to the tests. Testing took place between 7:00 and 9:00 a.m., and each subject's test time was fixed for the duration of the study (±30 ​min).

Blood samples were taken from the antecubital vein using sterile techniques. Venous blood samples were collected into serum separator tubes (8.5 ​mL Venosafe SST 2 advance, Becton Dickinson and Co., vacutainer, Plymouth, United Kingdom), which stood at room temperature for 30 ​min before being centrifuged for 10 ​min at 2200×*g*. After centrifugation, the supernatant was separated into microcentrifuge tubes and stored at −80 ​°C until further analysis. Serum insulin was determined using commercial chemiluminescence immunoassay techniques (Immulite 2000 XPi, Siemens Healthcare GmbH, Erlangen, Germany). Serum lipids, lipoproteins, and glucose were determined using KONELAB 20XTi analyser (Diagnostic Products Corporation, Los Angeles, CA, USA). Serum cytokines were analyzed with a 15-plex cytokine ELISA kit (#112433HU) according to the manufacturer's instructions using Quansys and Q-View software (Quansys Biosciences, Logan, UT, USA). Samples from the same participants were analyzed on the same ELISA plate. The detection limits and the inter- and intra-assay CVs for the cytokines were as follows: IL-1α 0.79 ​pg/ml, 9 ​%, 6 ​%; IL-2 0.008 ​pg/ml, 8 ​%, 6 ​%; IL-4 0.014 ​pg/ml, 11 ​%, 8 ​%; IL-5 0.28 ​pg/ml, 10 ​%, 8 ​%; IL-6 0.0059 ​pg/ml, 8 ​%, 6 ​%; IL-10 0.72 ​pg/ml, 11 ​%, 3 ​%; IL-13 0.1 ​pg/ml, 11 ​%, 5 ​%; IL-17 19.72 ​pg/ml, 9 ​%, 5 ​%; TNF-α 0.22 ​pg/ml, 12 ​%, 8 ​% and IFN-γ 0.22 ​pg/ml, 10 ​%, 5 ​%.”

To analyze the combined levels of cytokines related to anti-inflammatory (IL-4, IL-10, and IL-13) and pro-inflammatory (IFNγ, IL-1α, IL-6, IL-17, and TNFα) cytokines, the log transformed cytokine levels were expressed as Z-scores (mean 0, SD 1) within the whole study population. Summary pro-inflammatory and anti-inflammatory cytokine scores were calculated by averaging the Z-scores of the cytokines in each group. If the cytokine level was below the detection range, half of the detection range was imputed.

### 2.4 Knee MRI procedures and analysis

The T2 mapping (milliseconds), a parameter sensitive to collagen content and orientation and tissue hydration [33], was determined for the medial tibial articular cartilage. The knee with the higher degree of OA, as measured by the radiographic KL scale, was imaged. In cases where both knees had identical KL grades, the more symptomatic knee at the beginning of the study was assessed. Scans were conducted using a Siemens Magnetom Symphony Quantum 1.5-T system (Siemens AG, Medical Solutions, Erlangen, Germany) with a standard transmit/receive knee array coil using a sagittal multislice multiecho fast spin echo sequence (field of view 140 ​mm, acquisition matrix 256 ​× ​256, repetition time 2090 ​ms, eight echo times between 13 and 104 ​ms, echo train length 8, slice thickness 3 ​mm). Slices covering the central region of the medial tibial condyle were scanned with the leg stabilized to a fixed position. The weight-bearing cartilage regions of interest from the slices were manually segmented by two independent researchers (J.M., four years of experience and J.J., three years of experience) using an in-house MATLAB application (Mathworks, Inc., Natick, MA, USA). The interobserver error (CV_RMS_) for T2 full thickness regions of interest was on average 2 ​% in our laboratory [19], and the repeatability of the overall T2 measurement of the bulk medial tibial T2 in the short- and long-term has been found to be good [34]. The medial tibial condyle was selected as a point of interest, given that OA primarily impacts the medial compartment of the tibiofemoral joint [35,36]. Previous studies have shown that the severity of radiographic knee OA [37] and histological degeneration of cartilage [38] are associated with increased T2 values.

### 2.5 LTPA assessment

At baseline, an initial questionnaire was used to assess the physical activity levels of the participants prior to the commencement of the study. Throughout the intervention, all participants kept a diary recording the type, frequency, intensity, and duration of their LTPA. Activity that was carried out on a regular basis at least once a week with a minimum duration of 20 ​min was determined as LTPA. The participants in the exercise group reported any physical activity occurring outside the intervention. These data were sent to the research personnel monthly, and the gathered physical activity data, as well as the data from the initial questionnaire, were converted into metabolic equivalent task hours (MET-hours) per week using a scheme modified from Ainsworth et al. [39].

### 2.6 Exercise protocol

The assigned training frequency for the exercise group was three times a week for 12 months. Training included supervised 55-min high-impact, multidirectional aerobic, and step aerobics jumping exercise programs alternating every two weeks. Each exercise class consisted of a 15-min warm-up, 25 ​min of multidirectional, high-impact exercises, and 15-min cool down. The loading was gradually increased every 3 months by raising the height of the foam fences from 5 to 20 ​cm in aerobic exercises and the step benches from 10 to 20 ​cm in jumping exercises. Attendance record for each of the participants was kept by the instructors. The participants in the reference group were asked to maintain their usual activities and were provided the opportunity to attend a social group meeting every three months.

### 2.7 Statistical analysis

All analyses were conducted in accordance with the intention-to-treat principle. The data are presented as the means with standard deviations, medians with interquartile ranges or counts with percentages. Correlation coefficients between parameters were calculated by the Spearman method, using Sidak-adjusted probabilities. Statistical comparisons between groups were performed via t tests, chi-square tests or Mann–Whitney tests. Median changes in cytokine values between the baseline and 12-month measures were determined by Koch's nonparametric analysis of covariance. Multivariate linear regression analysis was used to identify the relationships between changes in the T2 of the medial tibial cartilage and LTPA MET-hours and changes in cytokine levels with the standardized regression coefficient Beta (β). Beta value is a measure of how strongly the predictor variable (cytokine levels) influences the criterion (MRI T2 or LTPA MET-hours) variable. The Beta is measured in units of standard deviation. Cohen's standard for Beta values above 0.10, 0.30 and 0.50 represents small, moderate and large relationships, respectively. Models were adjusted for age, smoking status, BMI, and baseline values of cartilage and LTPA MET-hours. Sidak-adjustment was used to assess the statistical significance of the tests. The assumptions of the regression models (normality and homoscedasticity of the residuals, heteroscedasticity, multicollinearity and model specification) were assessed using visual and statistical methods. Generalized estimating equations were used for the analysis of 12-month MET-hours. Normal distributions were evaluated graphically and with the Shapiro–Wilk W- test. Stata 18.0 (StataCorp LP; College Station, TX, USA) statistical package was used for the analysis.

## 3 Results

**Fig. 2:**
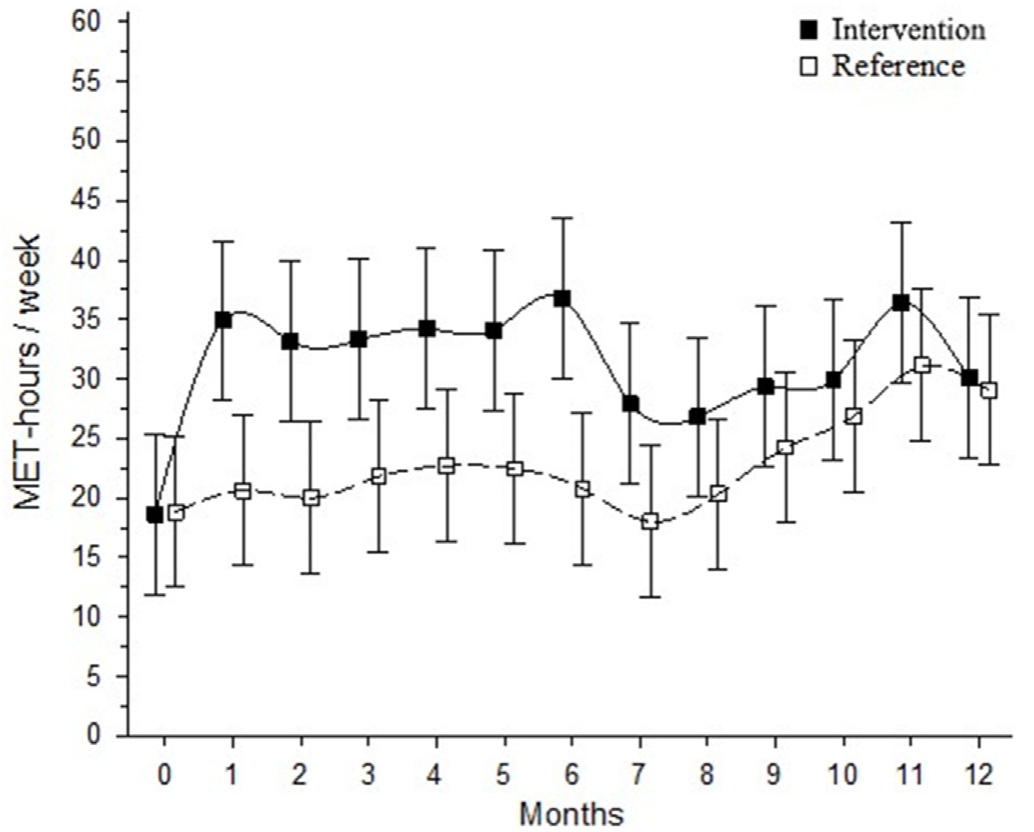
Weekly metabolic equivalent task hours by group with 95 ​% confidence intervals.

Two participants from the intervention group withdrew immediately after randomization due to their lack of time and dissatisfaction with their group allocation, and two others discontinued their participation at weeks 3 and 5. In total, four participants in the exercise group were lost to follow-up because they were not able to be reached (Fig. 1). Serum samples were not available from one exercise group participant and two reference group participants. Thus, 35 participants in the high-intensity exercise group and 38 participants in the reference group had valid and complete blood sample data and were included in the analysis. At baseline, participants in the reference group reported more LTPA and were shorter than those in the intervention group. Otherwise, the demographic and clinical characteristics of both groups were similar. The baseline characteristics are given in Table 1 and weekly LTPA MET-hours by group are presented in Fig. 2.

**Table 1:** Baseline demographic and clinical characteristics of the participants.

	Reference N ​= ​38	Exercise N ​= ​35
Age, mean (SD)	59 (4)	58 (4)
Height, cm, mean (SD)	162 (5)	166 (6)
Weight, kg, mean (SD)	69 (11)	72 (9)
Body mass index, kg/m^2^, mean (SD)	26.9 (4.1)	27.1 (2.9)
Current smokers, n (%)	3 (8)	1 (3)
Alcohol consumption, n (%)	35 (92)	33 (94)
Education years, mean (SD)	12.3 (3.4)	13.5 (3.4)
Kellgren Lawrence grade, n (%)		
1	13 (34)	11 (31)
2	25 (66)	24 (69)
WOMAC (range, 0–100), mean (SD)		
Pain	6.4 (5.9)	7.4 (8.3)
Stiffness	9.2 (9.4)	10.7 (13.0)
Physical function	3.6 (3.7)	4.4 (5.1)
NSAID usage, n (%)	17 (45)	21 (60)
Knee pain, VAS, mm, mean (SD)	9 (11)	11 (14)
Leisure-time physical activity^a^, MET hours/week, mean (SD)	23.2 (16.1)	15.3 (10.9)
Diseases, n (%)		
High blood pressure	10 (26)	9 (26)
Lung condition	3 (8)	5 (14)
Neurologic condition	2 (5)	0 (0)

WOMAC = Western Ontario and McMaster; Universities Osteoarthritis Index; MET-hour ​= ​metabolic equivalent task hour; NSAID ​= ​non-steroidal anti-inflammatory drug; VAS = Visual analogue scale.

a Prior to the commencement of the study.

There were six medical consultations in the high-impact exercise intervention group, which included musculoskeletal injuries and other symptoms such as knee swelling, Achilles tendon pain, hamstring distension, low back pain, ankle sprain, and asthma-like symptoms. Every trainee resumed their exercise regimen within five to twenty-one days. Two reference participants required a medical consultation due to a cardiac dysrhythmia and previous meniscal tear injury. In the exercise group, the mean training compliance, measured by attendance at all training sessions offered, was 68 ​% and the mean weekly training frequency was 2.1 (SD 0.9) [19].

### 3.1 Cytokine measurements

The baseline values and treatment effects of the cytokines are given in Table 2. No significant differences in the changes of any combinatory or individual cytokine levels were observed between the groups after the 12-month intervention. However, there were significant within-group changes in the IL-13 levels in the reference (0.58 ​pg/ml; 95 ​% CI 0.07 to 1.30) and exercise groups (0.31 ​pg/ml; 95 ​% CI 0.09 to 1.89).

**Table 2:** Baseline and change in cytokine levels after the treatment.

	Baseline		Change to 12 months		*P*-value^b^	
	Reference n ​= ​38 Median (IQR)	Exercise n ​= ​35 Median (IQR)	Reference n ​= ​38 Median (95 ​% CI)^a^	Exercise n ​= ​35 Median (95 ​% CI)^a^	Crude	Adjusted^c^
Pro-inflammatory Z-score	−0.08 (−0.36 to 0.41)	−0.09 (−0.47 to 0.36)	0.20 (−0.50 to 0.50)	0.00 (−0.60 to 0.60)	0.63	0.58
Anti-inflammatory Z-score	0.13 (−0.11 to 0.35)	0.04 (−0.39 to 0.21)	0.10 (−0.50 to 0.50)	−0.20 (−0.60 to 0.90)	0.89	0.87
IL1α, pg/ml	13.90 (10.70–21.30)	13.90 (7.20–21.20)	−1.90 (−7.80 to 3.70)	−0.10 (−3.50 to 5.70)	0.81	0.91
IL-2, pg/ml	2.81 (2.23–4.46)	3.53 (2.40–5.19)	−0.37 (−1.38 to 1.29)	−0.03 (−1.76 to 0.35)	0.78	0.57
IL-4, pg/ml	0.07 (0.05–0.11)	0.07 (0.05–0.10)	0.01 (−0.01 to 0.07)	0.01 (−0.02 to 0.01)	0.33	0.26
IL-5, pg/ml	0.92 (0.14–1.74)	0.63 (0.14–1.78)	0.02 (−0.40 to 0.61)	0.00 (−0.20 to 0.22)	0.45	0.10
IL-6, pg/ml	2.61 (2.06–3.37)	2.53 (1.73–4.39)	0.10 (−1.04 to 0.49)	0.06 (−1.23 to 0.83)	0.95	0.97
IL-10, pg/ml	19.20 (14.50–21.90)	17.30 (15.10–21.30)	−0.10 (−3.60 to 3.50)	−0.10 (−4.10 to 4.30)	0.56	0.82
IL-13, pg/ml	2.87 (1.93–4.84)	3.09 (1.81–4.91)	0.58 (0.07–1.30)	0.31 (0.09–1.89)	0.79	0.80
IL-17, pg/ml	26.50 (20.20–52.70)	42.40 (21.80–65.00)	1.60 (−12.20 to 7.80)	−6.40 (−22.10 to 0.70)	0.09	0.39
IFN-γ, pg/ml	3.67 (2.77–6.21)	4.68 (2.70–6.06)	0.65 (−0.77 to 1.56)	−0.90 (−1.96 to 2.50)	0.39	0.40
TNF-α, pg/ml	6.35 (3.35–8.59)	6.22 (0.11–8.35)	0.78 (−0.35 to 1.86)	0.00 (−0.20 to 5.49)	0.65	0.39

a Confidence intervals calculated using bias corrected bootstrapping.

b P-values for differences in change between the intervention and reference groups.

c Adjusted by baseline values.

### 3.2 Relationships between cytokine levels and MRI T2 and weekly LTPA MET-hours

Correlations between cytokine levels and articular cartilage quality, as determined by T2 mapping of the medial tibial condyle and LTPA MET-hours at baseline, are shown in Table 3. The correlation analysis of the entire study group (n ​= ​73) revealed that there were no associations between cytokine levels and the T2 relaxation time of medial tibial cartilage. However, the IL-10 level was correlated with the number of LTPA MET-hours (r ​= ​0.36; 95 ​% CI 0.03 to 0.62).

**Table 3:** Correlations between the cytokine levels and medial tibial condyle T2 and weekly MET-hours at baseline (n ​= ​73).

	T2 tibia med. r (95 ​% CI)	MET-hours/week. r (95 ​% CI)
Pro-inflammatory	0.04 (−0.29 to 0.36)	0.05 (−0.28 to 0.38)
Anti-inflammatory	−0.12 (−0.43 to 0.22)	−0.01 (−0.34 to 0.32)
IL-1α	0.08 (−0.26 to 0.40)	−0.03 (−0.36 to 0.31)
IL-2	−0.12 (−0.43 to 0.22)	0.21 (−0.13 to 0.51)
IL-4	0.10 (−0.24 to 0.41)	−0.25 (−0.54 to 0.09)
IL-5	0.09 (−0.25 to 0.41)	−0.08 (−0.40 to 0.25)
IL-6	0.04 (−0.29 to 0.36)	0.07 (−0.27 to 0.39)
IL-10	−0.20 (−0.50 to 0.14)	0.36 (0.03–0.62)∗
IL-13	−0.21 (−0.50 to 0.13)	−0.12 (−0.44 to 0.22)
IL-17	0.11 (−0.22 to 0.43)	0.12 (−0.22 to 0.44)
IFN-γ	0.03 (−0.31 to 0.35)	−0.01 (−0.34 to 0.32)
TNF-α	−0.12 (−0.43 to 0.22)	0.06 (−0.28 to 0.38)

T2 tibia med. ​= ​transverse relaxation time of the medial tibial condyle; MET-hour ​= ​metabolic equivalent task hour, Sidak-adjusted probabilities: ∗p ​< ​0.05.

**Fig. 3:**
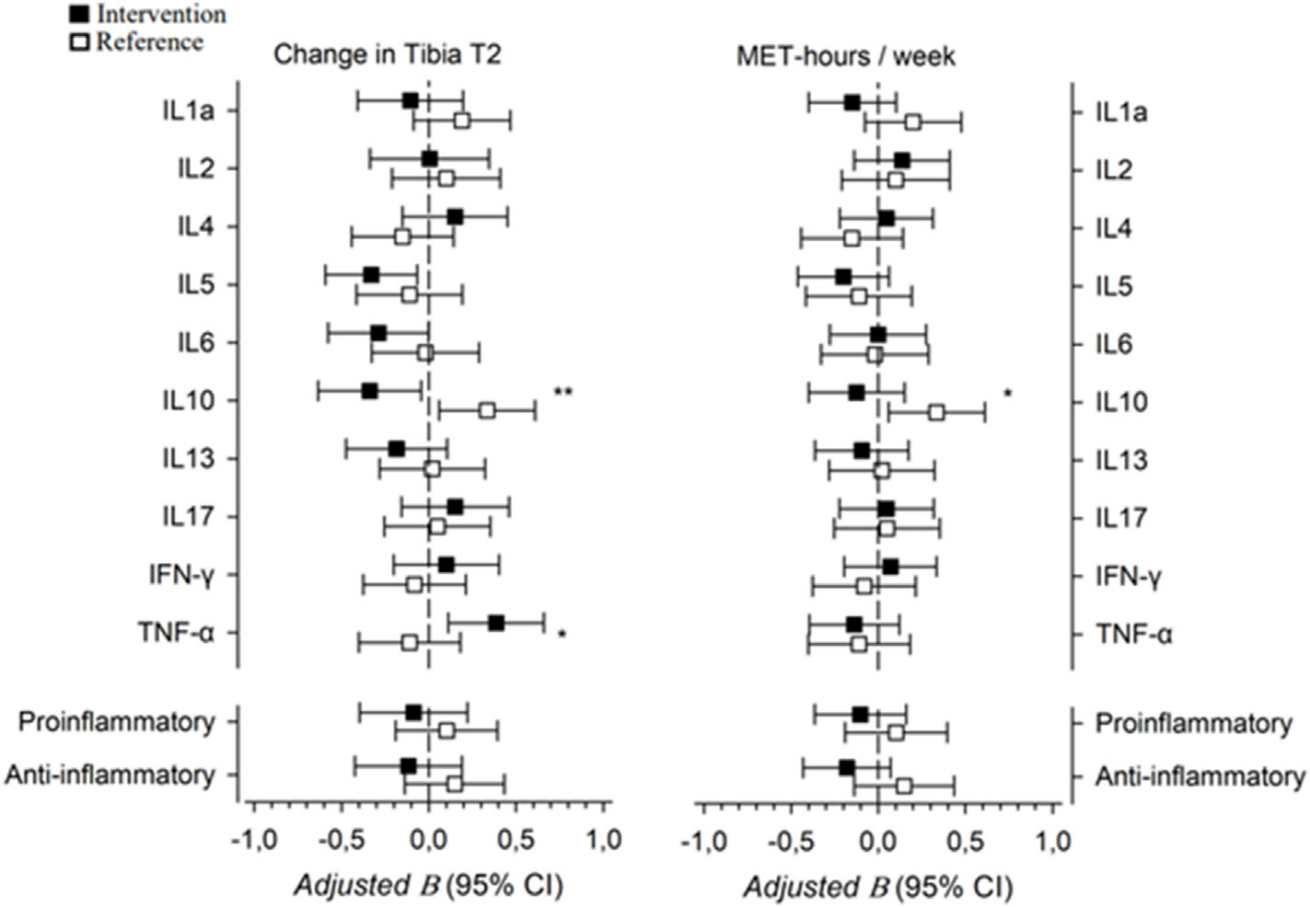
Associations between changes in cytokine levels and medial tibial condyle T2 and weekly metabolic equivalent task hours by group. The results presented have not been adjusted for multiple comparisons. Significant crude p-values for between group differences are presented. ∗p ​< ​0.05, ∗∗p ​< ​0.01.

A multivariable linear regression was used to ascertain the relationships between changes in cytokine levels and T2 of the medial tibial condyle and LTPA MET-hours. In the intervention group, changes in TNF-α levels were significantly associated with changes in the medial tibial condyle T2 at 12 months (B ​= ​0.39; 95 ​% CI 0.11 to 0.66; R^2^ ​= ​0.32). In the reference group, changes in IL-10 levels were significantly associated with changes in the medial tibial condyle T2 (B ​= ​0.34; 95 ​% CI 0.06 to 0.61; R^2^ ​= ​0.38) and weekly LTPA MET-hours (B ​= ​0.34; 95 ​% CI 0.08 to 0.63; R^2^ ​= ​0.44). The model's beta coefficients for the changes in the medial tibial condyle and weekly LTPA MET-hours are shown in Fig. 3.

## 4 Discussion

The aim of this secondary analysis was to investigate the effects of a 12-month high-impact training program compared with a reference group on systemic cytokine levels in postmenopausal women with mild knee OA. The findings of this study indicate that progressively implemented high-impact exercise did not result in any significant changes in serum cytokines. These results corroborate our previous findings from the same study, which demonstrated that high-impact exercise did not have any detrimental effects on tibiofemoral articular cartilage or result in significant changes in perceived pain, stiffness or self-rated physical function as assessed by the Western Ontario and McMaster Universities Osteoarthritis Index in either group [19]. In contrast, training improved the quality of the patellar cartilage while improving femoral neck strength and increasing bone mineral mass [[19], [20], [21]]. Weekly LTPA MET-hours, determined from the physical activity diaries, were greater in the exercise group, especially during the first half of the intervention. These findings are consistent with those of Hartley et al. [22] who also reported that high-impact training is not detrimental to knee articular cartilage but increases femoral neck bone mineral mass and strength compared with those of the control leg. Therefore, the current results and those of previous studies [[19], [20], [21], [22]] suggest that high-impact exercise may be a feasible exercise modality for postmenopausal women with knee OA who are also at risk of osteoporosis and thus could benefit from bone favorable exercise. To the best of our knowledge, this is the first study to investigate the effects of high-impact training on serum cytokine levels in participants with knee OA.

Previous studies have reported varying results on the associations between different physical exercise interventions and their effects on cytokine levels in OA. Our findings are consistent with the meta-analyses by Puts et al. [29] and Bricca et al. [40] which found that aerobic and strength exercise regimens did not significantly affect IL-6 or TNF levels in people with or at risk of knee OA. Likewise, Scuhlz et al. [28] reached a similar conclusion in their systematic review, which included aerobic exercise regimens. Additionally, high or low intensity strength training three times a week for 18 months did not affect serum IL-6 levels, nor did facility-based individualized exercise therapy three times a week for 12 weeks have any effect on plasma IL-6 or IL-10 levels in knee OA patients compared with controls [41,42]. In contrast, some studies have reported significant reductions in IL-6 levels but not other cytokine levels after 12 weeks of swimming, cycling, or combined flexibility and strength training regimens in OA patients [43,44]. Overall, exercise in OA patients seems to have limited anti-inflammatory, if any, effects on inflammatory cytokines [28,29,[40], [41], [42], [43], [44]].

To complement the analysis, we observed associations between changes in cytokine levels and medial tibial T2 values and weekly LTPA. A moderate relationship was observed between the IL-10 levels and T2 of the medial tibial cartilage, with an increase in IL-10 levels being associated with increased T2 of the medial tibial cartilage in the reference group but not in the exercise group. Additionally, in the exercise group, increases in TNF-α levels were moderately associated with increased T2 of the medial tibial cartilage. TNF-α is considered to be a major player affecting OA-related inflammatory progression, as it inhibits chondrogenesis and promotes the release of pro-inflammatory cytokines and matrix metalloproteinases [24]. In studies with a two-to three-year follow-up period, TNF-α levels were found to be associated with the radiological progression of knee OA and a change in cartilage volume [45,46]; however, Pan et al. [47] did not identify such an association in their longer follow-up period. Regarding IL-10, Botha-Scheepers et al. [46] found that OA patients in the highest quartile of IL-10 production had a fourfold increased risk of joint space narrowing progression compared with those in the lowest quartile.

In our study, changes in IL-6 levels were not associated with changes in T2 of the medial tibial condyle. This finding is somewhat contradictory to earlier findings, where IL-6 levels were found to be associated with cartilage volume loss in the medial and lateral compartments of the tibia [45,47]. However, it should be noted that we examined tibial cartilage quality instead of cartilage volume. In addition, circulatory IL-6 levels have been shown to be an independent predictor of the development of knee OA [48]. Although the intervention had no effect on the IL-10 levels, an association between the IL-10 levels and LTPA was observed in the reference group. This could indicate a possible association between certain types of LTPA and IL-10 levels. However, the relationship between IL-10 and exercise remains complex in people with OA, especially in the presence of different identified clinical phenotypes [49].

This study has several strengths. All the exercise sessions were conducted by experienced exercise instructors, the overall physical activity of the participants was recorded throughout the trial using LTPA diaries, the intervention duration was sufficient, and training compliance was high. The study design fulfilled all the important quality criteria of an RCT with the exception of blinding of the participants, which is practically unachievable in an exercise therapy such as this one [50]. This study has several limitations, one of which is that the variables considered in this secondary analysis were not apparent in the original trial's registration; therefore the analysis was performed only on the available samples. However, given the paucity of published studies assessing the effects of intensive training programs on systemic cytokine levels in postmenopausal women with mild knee OA, the findings of this study are still relevant. It should be noted that as the cytokine levels were assessed from blood samples which reflect changes in systemic inflammation, these levels may not exclusively reflect changes related to OA or knee joint-specific changes. Changes in T2 of the medial tibial cartilage were assessed using single sagittal slice covering the regions of interest and the segmentation procedure inevitably involves some degree of subjectivity. However, the researchers performing the segmentation underwent extensive training and demonstrated excellent interobserver reliability. Additionally, T2 mapping is well-established and sensitive method, particularly for detecting changes in collagen content and orientation [33]. Although the high-impact exercise regimen was well tolerated and showed no adverse effects on systemic cytokine levels or the biochemical composition of knee cartilage after 12 months, further research is required to determine its long-term effects. Furthermore, since these results focused only on women with mild knee OA, future intensive training programs should focus on individuals with moderate stage knee OA who are also at risk of osteoporosis.

In conclusion, this study revealed that a progressive high-impact exercise regimen did not affect systemic cytokine levels compared to the reference group and could therefore offer a possible mode of exercise for postmenopausal women with mild knee OA.

## Author contributions

The study was conceived and designed by AH, MTN and TJ. The trial was conducted and supervised by AH, MTN, SP, JM and JKI. The first manuscript was drafted by VMK. All authors participated in critical revision of the article for important intellectual content and approved the manuscript for submission. HK provided statistical expertise. All the authors participated in the study data analysis and interpretation.

## CRediT author statement

Ville-Markus Konola: Writing - Original Draft.

Juhani Multanen: Writing - Review & Editing, Project administration, Investigation.

Johanna K. Ihalainen: Writing - Review & Editing, Methodology.

Jukka E. Hintikka: Writing - Review & Editing, Investigation.

Timo Jämsä: Writing - Review & Editing, Conceptualization.

Hannu Kautiainen: Writing - Review & Editing, Formal analysis, Visualization.

Miika T. Nieminen: Writing - Review & Editing, Conceptualization, Methodology.

Satu Pekkala: Writing - Review & Editing, Investigation, Methodology.

Maarit Valtonen: Writing - Review & Editing.

Ari Heinonen: Supervision, Writing - Review & Editing, Conceptualization, Funding acquisition.

## Role of the funding source

This study was supported by the Research Council of Finland (Dnro 123140 and 122276), the Finnish Ministry of Education and Culture (Dnro 70/627/2008, Dnro 46/627/2009, Dnro 49/627/2010), the Yrjö Jahnsson Foundation, the Finnish Cultural Fund and the 10.13039/501100022768Maire Lisko Foundation. None of the sponsors above were involved in the study design; the collection, analysis, and interpretation of the data; the writing of the manuscript; or the decision to submit the manuscript for publication.

## Declaration of competing interest

All authors state that they have no conflicts of interest.
